# Long-term efficacy of CDK4/6 inhibitors in early HR+, HER2- high-risk breast cancer: An updated systematic review and meta-analysis

**DOI:** 10.3389/fphar.2025.1564437

**Published:** 2025-07-11

**Authors:** Ying Liu, Jun Su, Ping Wu, Wenjie Lv

**Affiliations:** ^1^ Department of Breast Surgery, Xinhua Hospital Affiliated to Shanghai Jiao Tong University School of Medicine, Shanghai, China; ^2^ Department of Oncology, Xinhua Hospital Affiliated to Shanghai Jiao Tong University School of Medicine, Shanghai, China

**Keywords:** CDK4/6 inhibitor, early breast cancer, meta-analysis, follow-up duration, iDFS

## Abstract

**Background:**

The confirmed efficacy of combining CDK4/6 inhibitors with endocrine therapy (ET) in HR+, HER2− early breast cancer patients in longer follow-up necessitates further investigation. This meta-analysis aims to comprehensively assess the impact of follow-up duration on the therapeutic activity in high-risk patients.

**Methods:**

We systematically searched PubMed, Cochrane Library, Embase, Medline, Web of Science, and relevant conference abstracts up to 19 October 2024. The included RCTs examined CDK4/6 inhibitors with ET in HR + HER2− early breast cancer patients. Hazard ratios (HR) with 95% confidence intervals (CI) were calculated to analyze invasive disease-free survival (iDFS).

**Results:**

Four RCTs with 3-year and 4-year iDFS data (n = 17,749) were included. The results showed that the CDK4/6 inhibitor group had significantly better iDFS compared to the ET group at both 3 years (HR = 0.81, 95% CI 0.70–0.94, P = 0.006) and 4 years (HR = 0.78, 95% CI 0.66–0.94, P = 0.007). Subgroup analysis revealed that in LN + patients, the CDK4/6 inhibitor group also had significantly better iDFS at both 3 years (HR = 0.84, 95% CI 0.73–0.97, P = 0.02) and 4 years (HR = 0.74, 95% CI 0.68–0.81, P < 0.00001). For N0 patients, iDFS benefits became more evident over time. At 4 years, combination therapy showed significant improvement (HR = 0.65, 95% CI 0.45–0.94, P = 0.02). For stage II patients, CDK4/6 inhibitors combined with ET showed no statistically significant difference in iDFS at treatment completion (HR = 0.78, 95% CI 0.59–1.05, P = 0.10), but significantly improved iDFS at 4 years (HR = 0.66, 95% CI 0.50–0.87, P = 0.003).

**Conclusion:**

The iDFS improvement with CDK4/6 inhibitors becomes more evident over time, suggesting broader benefits and enhanced long-term efficacy for HR+, HER2-breast cancer patients especially with N0 or Stage II.

**Systematic Review Registration:**

PROSPERO database, CRD42024593864.

## Highlights

CDK4/6 inhibitors combined with endocrine therapy continue to improve invasive disease-free survival (iDFS) in HR+, HER2-early breast cancer with longer follow-up.

CDK4/6 inhibitors could benefit a broader population and enhance long-term efficacy for HR+, HER2-breast cancer patients, including those with N0 or Stage II disease.

The survival benefit of CDK4/6 inhibitors is partly attributed to the reduction in long-term recurrence.

## Introduction

Hormone receptor (HR)-positive, human epidermal growth factor receptor 2(HER2)-negative early breast cancer accounts for nearly 70% of all breast cancers and generally has a favorable prognosis (Giaquinto et al., 2022). However, a significant portion of these patients remain at risk for recurrence, particularly those with high-risk features such as lymph node involvement and genomic risk factors, highlighting the ongoing need for improved treatments.

Cyclin-dependent kinases 4 and 6 (CDK4/6) inhibitors targeted key regulators of cell cycle, blocking progression through the G1 phase (Piezzo et al., 2020). CDK4 and CDK6 binds to Cyclin D, leading to the continuous phosphorylation and functional inactivation of the retinoblastoma protein. This process weakens retinoblastoma protein’s function to inhibit cell cycle progression, facilitating the transition from the G1 to the S phase. The Cyclin D-CDK4/6 pathway plays a crucial role in the proliferation and survival of tumors, including breast cancer. Inhibitors targeting CDK4/6, as palbociclib, ribociclib, and abemaciclib offer both hope and challenges for the treatment of breast cancer (Besson et al., 2008; Bai et al., 2023; Wu et al., 2022).

Currently, CDK4/6 inhibitors are the first-line treatment for HR+, HER2-, advanced breast cancer, with both abemaciclib and ribociclib receiving FDA approval for use in early breast cancer (Morrison et al., 2024). Abemaciclib with endocrine therapy (ET) was approved for the adjuvant treatment of adult patients with HR+, HER2-, node-positive, early breast cancer at high risk of recurrence (FDA, 2023). The established benefit observed in early breast cancer prompted the investigation of CDK4/6 inhibitors in a boarder population. Notably, 4 years follow up data from the NATALEE trial, announced at the 2024 ESMO conference, showed that the benefit in N0 patients began to emerge compared with the 3-year data. (Fasching et al., 2024). Almost at the same time, FDA approved ribociclib with an aromatase inhibitor for the adjuvant treatment of adults with HR+, HER2-, stage II and III early breast cancer at high risk of recurrence (FDA, 2024). This approval broadened the drug’s coverage to N0 patients not only LN + patients. These impressive results raise the question of whether CDK4/6 inhibitors should be widely used to reduce long-term recurrence in HR + early breast cancer patients. This study aims to explore the impact of follow-up duration on the efficacy of CDK4/6 inhibitors in HR+, HER2-breast cancer patients in the early setting, especially in high-risk N0 or Stage II patients.

## Methods

### Study objectives

The objective of this meta-analysis was to investigate the impact of treatment duration on the efficacy of CDK4/6 inhibitors combined with endocrine therapy (ET) versus ET alone in early-stage, HR+, HER2-breast cancer. The primary endpoint was invasive disease-free survival (iDFS) across different treatment durations. All time-to-event endpoints were defined according to the standardized definitions for efficacy endpoints (STEEP) criteria.

Subgroup analyses were conducted to evaluate iDFS based on patients’ clinical stages, including N0 and Stage II, which are typically excluded from clinical trials for intensive adjuvant therapy in HR + HER2-patients.

### Search strategy

This systematic review and meta-analysis was conducted in accordance with Preferred Reporting Items for Systematic Reviews and Meta-Analyses (PRISMA)guidelines (Page et al., 2021). A comprehensive search of the following databases was performed: PubMed, Cochrane Library, Embase, Medline, Web of Science, as well as meeting abstracts and conference proceedings from the ESMO, ASCO, and SABCS websites. All studies were screened from database inception to 19 October 2024. Keywords used in search strategy included “CDK4/6 inhibitors” “Palbociclib”, “Ribociclib”, “Abemaciclib”, “Dalpiciclib”, “breast cancer”, “adjuvant therapy”, “randomization”. Specific keywords and free text terms were combined with Boolean operators. The review was registered in the international prospective register of systematic reviews (PROSPERO database CRD42024593864).

### Data extraction and synthesis

We selected randomized controlled trials (RCTs) based on the PICO criteria: (1) patients pathologically diagnosed with early-stage, HR+, HER2-breast cancer; (2) intervention: CDK4/6 inhibitor plus endocrine therapy (ET); (3) control: ET or placebo plus ET; (4) reported invasive disease-free survival (iDFS) with associated hazard ratios (HR). Phase I/II clinical trials, single-arm trials, non-RCTs, systematic reviews, and studies with unavailable data were excluded. Data from the eligible RCTs were extracted by 2 reviewers (YL and PW), any discrepancies were solved by discussion with a third author (WL). This study was carried out using the Review Manager software (version 5.4).

### Statistical analysis

Review Manager software (version 5.4) was used for all statistical analyses and the generation of forest plots. Engauge Digitizer (version 12.1) was employed to extract data from Kaplan-Meier survival curves provided in the trials. Hazard ratios (HR) and 95% confidence intervals (CI) were calculated to assess the impact of CDK4/6 inhibitors combined with endocrine therapy (ET) on invasive disease-free survival (iDFS). An HR < 1 indicates improved iDFS with CDK4/6 inhibitors and ET, while an HR > 1 indicates reduced iDFS with CDK4/6 inhibitors and ET. Statistical significance was set at P < 0.05.

Cochran’s Q and I^2^ tests were used to evaluate heterogeneity among the included studies. A Cochran’s Q statistic of P < 0.1 or an I^2^ of ≥50% indicated significant heterogeneity. In such cases, a random-effects model was applied. Conversely, a fixed-effects model was used when Cochran’s Q statistic was >0.1 and I^2^ was <50%.

## Results

**FIGURE 1:**
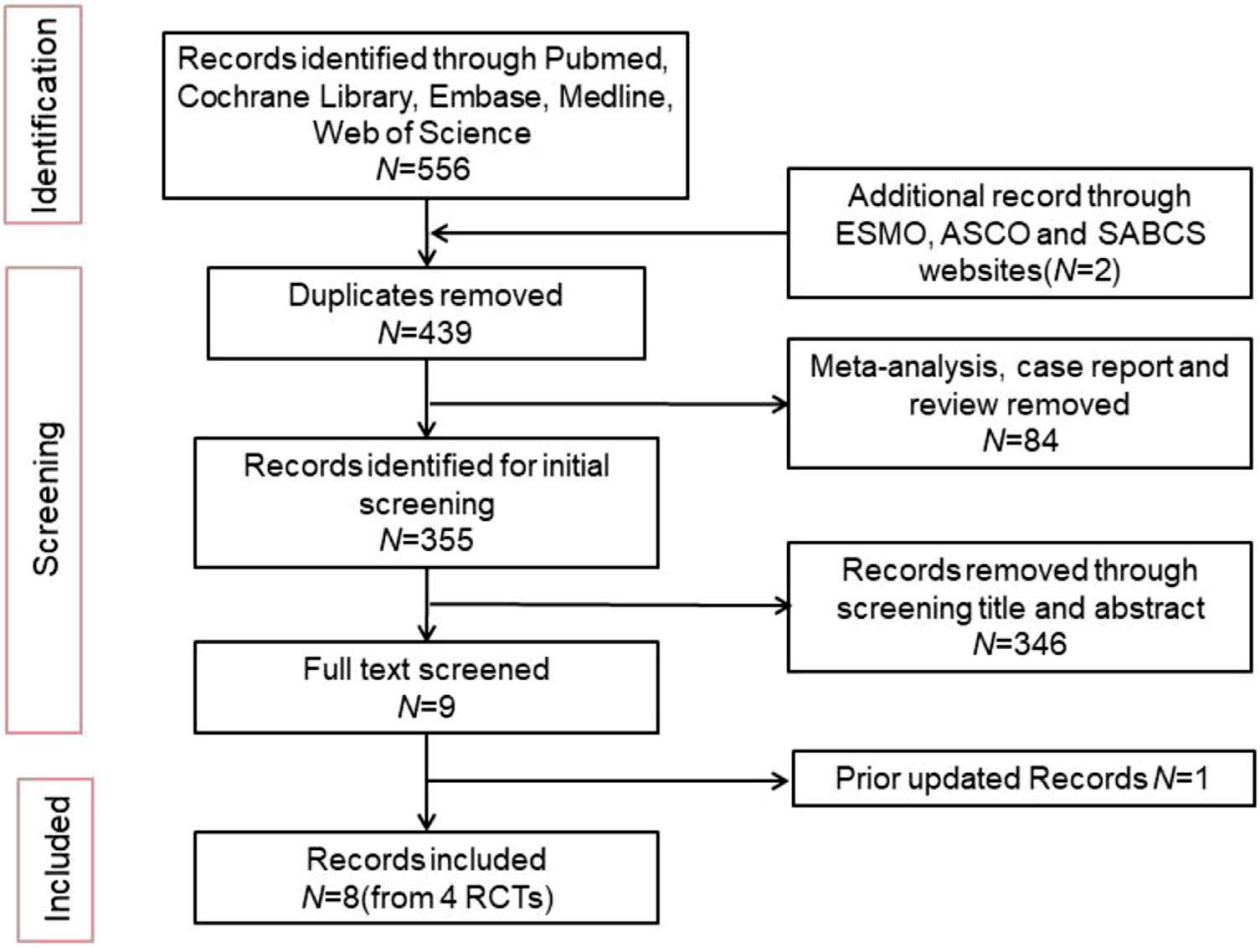
The flowchart of process for the study.

A total of 556 articles were retrieved from the literature search, and 8 articles were included from 4 clinical trials (Figure 1). The latest updated results from the NATALEE and Penelope-B trials were obtained from ESMO 2024 (Fasching et al., 2024; Loibl et al., 2025). We excluded 117 duplicate articles and 84 articles that were meta-analyses, case reports, or reviews. An initial review of 355 articles was conducted, and 346 articles were excluded for not meeting the inclusion criteria based on their titles and abstracts. The full text of 9 articles was screened, and 1 article was excluded because the updated data did not meet the inclusion criteria. Ultimately, 8 articles were included. The following reports were included in the primary analysis: Penelope-B trial (Loibl et al., 2025; Loibl et al., 2021), PALLAS trial (Gnant et al., 2022; Mayer et al., 2021), NATALEE trial (Fasching et al., 2024; Slamon et al., 2024), monarchE trial (Johnston et al., 2023; Johnston et al., 2020). Since the hazard ratios (HR) were not published in the 3-year data of the monarchE trial and the 4-year data of the Penelope-B trial, data were extracted from Kaplan-Meier (KM) curves for the meta-analysis. Table 1 shows the characteristics of the included studies.

**TABLE 1:** Studis included in the present meta-analysis.

Study	NCT number	Study design	Phase	Patients	Gender	Treatment	CDK4/6I treatment period (years)	number (e/c)	Endpoints	Follow-up time (months)	Time of iDFS
PALLAS	NCT02513394	RCT	Phase III	Patients with histologically confirmed stage II or III; HR+ and HER2- breast cancer	female and male	Palbociclib + ET ± OFS vs. et alone ± OFS	2	5,761 (2,884/2,877)	iDFS; invasive BC-free survival; LLRFS; DRFS; OS; AEs	31	4 years
NATALEE	NCT03701334	RCT	Phase III	Patients with HR+, HER2- early BC with anatomical stage IIA:N0 and G3; N0 and G2 and Ki-67 ≥ 20% or high genomic risk; N1+; IIB or III	female and male	Ribociclib + NSAI ± goserelin VS NSAI ± goserelin	3	5,101 (2,549/2,552)	iDFS; DDFS; RFS; OS; Aes; QoL	44.2	4 years
MonarchE	NCT03155997	RCT	Phase III	Patients with (1) ≥4 pALNs or (2)1–3 pALNs with additional high-risk features: G3,T ≥ 5 cm,or Ki-67 ≥ 20%	female	Abemaciclib + ET vs. ET	2	5,637 (2,808/2,829)	iDFS; OS; DRFS; safety	54	5 years
Penelope-B	NCT01864746	RCT	Phase III	women with HR+,HER2- primary BC; non-pCR after taxane-containing NACT; CPS-EG score ≥3 or 2 and ypN1	female	Palbociclib + ET vs. placebo + ET	1	1,250 (631/619)	iDFS; DDFS; OS; LRRFI; safety	77.8	6-year

e, experiment group; c, control group; BC, breast cancer; pALNs, pathologic axillary lymph nodes; CPS-EG, clinical pathological staging-estrogen receptor grading score; LRRFI, locoregional relapse-free interval; DDFS, distant disease-free survival; DRFS, distant relapse-free survival; LRRFS, loco-regional recurrence-free survival; RFS, recurrence-free survival; Qol, quality of life; Aes, adverse events.

**FIGURE 2:**
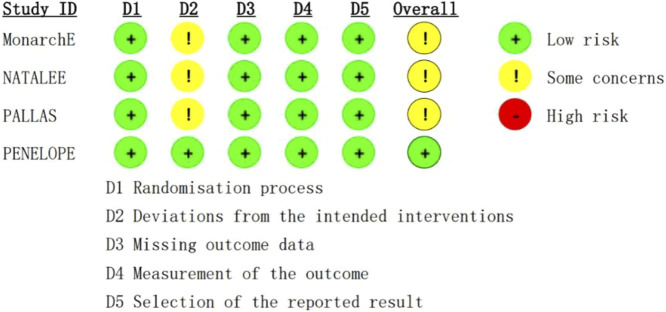
The bias risk assessment.

A total of 17,749 patients were enrolled in this study, 8,872 were treated with CDK4/6 inhibitors plus ET, and 8,877 were treated with ET. The characteristics of population included in this study is summarized in Table 2. The evaluation of the risk bias using ROB2 is shown in Figure 2. The overall rating indicates “some concerns”, largely attributable to the open-label design of three included trials, where deviations from intended interventions couldn't be fully determined. Allocation concealment was similarly rated “some concerns”.

**TABLE 2:** Characteristics of patients in clinical trials.

Study	MonarchE		Penelope-B		PALLAS		NATALEE	
Group	Abemaciclib + ET	ET	Palbociclib + ET	ET	Palbociclib + ET	ET	Ribociclib + ET	ET
N	2,808	2,829	631	619	2,884	2,877	2,549	2,552
Age, median, (range)	51 (44–60)	51 (44–60)	49 (22–76)	48 (19–79)	52 (25–90)	52 (22–85)	52 (24–90)	52 (24–89)
Stage, n(%)								
Ⅰ or ⅡA	324 (11.5)	353 (12.5)	—		513 (17.8)	519 (18.0)	588 (23.1)	526 (20.6)
ⅡB or Ⅲ	2,371 (84.4)	2,376 (84.0)			2,370 (82.2)	2,358 (82.0)	2060 (80.8)	2025 (79.3)
N stage, n(%)								
N0	0	0	66 (10.5)	71 (11.5)	365 (12.7)	385 (13.4)	285 (11.2)	328 (12.9)
N1	1,118 (40)	1,142 (40)	433 (68.6)	417 (67.4)	1,431 (49.6)	1,411 (49.0)	2,261 (88.7)	2,219 (87.0)
N2 or N3	1,682 (60.0)	1,680 (59.4)	132 (20.9)	131 (21.2))	1,086 (37.7)	1,081 (37.6)		
Grade, n(%)								
1 or 2	1,586 (56.5)	1,611 (56.9)	386 (61.2)	366 (59.1)	1926 (66.8)	1971 (68.5)	1,676 (65.8)	1,691 (66.3)
3	1,086 (38.7)	1,064 (37.6)	237 (37.6)	245 (39.6)	836 (29.0)	769 (26.7)	521 (20.4)	549 (21.5)
Menopausal status, n(%)								
Premenopausal women	1,221 (43.5)	1,232 (43.5)	300 (47.5)	316 (51.1)	1,303 (45.2)	1,323 (46.0)	1,115 (43.7)	1,123 (44.0)
Postmenopausal women	1,587 (56.5)	1,597 (56.5)	331 (52.5)	303 (48.9)	1,562 (54.2)	1,534 (53.3)	1,423 (55.8)	1,420 (55.6)
Men	0	0	0	0	17 (0.6)	19 (0.7)	11 (0.4)	9 (0.4)
Neoadjuvant or adjuvant chemotherapy, n(%)								
Chemotherapy	1,642 (58.5)	1,647 (58.2)	0	0	1,448 (50.2)	1,427 (49.6)	1,223 (48.0)	1,220 (47.8)
Neoadjuvant chemotherapy	1,039 (37.0)	1,048 (37.0)	631	619	965 (33.5)	974 (33.9)	1,085 (42.6)	1,095 (42.9)
Off CDK4/6 treatment, n(%)								
3-year analysis	—		100		74.8		54	
4-year analysis	81.3		—		87.9		100	
Complete CDK4/6 treatment, n(%)								
3-year analysis	—		80.5		32.3		20.2	
4-year analysis	69		—		44.9		62.8	

—indicates that the data is not provided.

### IDFS of all patients

**FIGURE 3:**
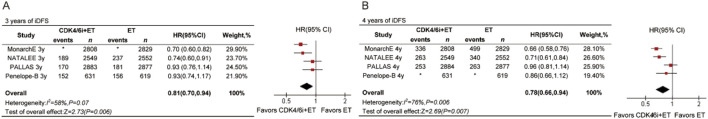
Forest plots of pooled hazard rations for 3-year iDFS **(A)** and 4-year iDFS **(B)** of CDK4/6 inhibitors plus ET versus ET. *Data are not provided.

The results demonstrated that the CDK4/6 inhibitors group achieved superior invasive disease-free survival (iDFS) compared to the endocrine therapy (ET) group. This difference was statistically significant at both the 3-year follow-up (HR = 0.81, 95% CI 0.70–0.94, P = 0.006, Figure 3A) and the 4-year follow-up (HR = 0.78, 95% CI 0.66–0.94, P = 0.007, Figure 3B). Notably, the benefits for patients increased over time, as reflected by the progressively lower HR values with extended follow-up periods.

Among the four studies, NATALEE and PALLAS included both lymph node-positive and lymph node-negative patients, with follow-up data available for 3 and 4 years. MonarchE included only lymph node-positive patients and provided 4-year follow-up data. Both MonarchE and NATALEE included data for Stage II and Stage III patients. Subgroup analyses were conducted based on lymph node status and AJCC stage using these data.

### Nodal status

**FIGURE 4:**
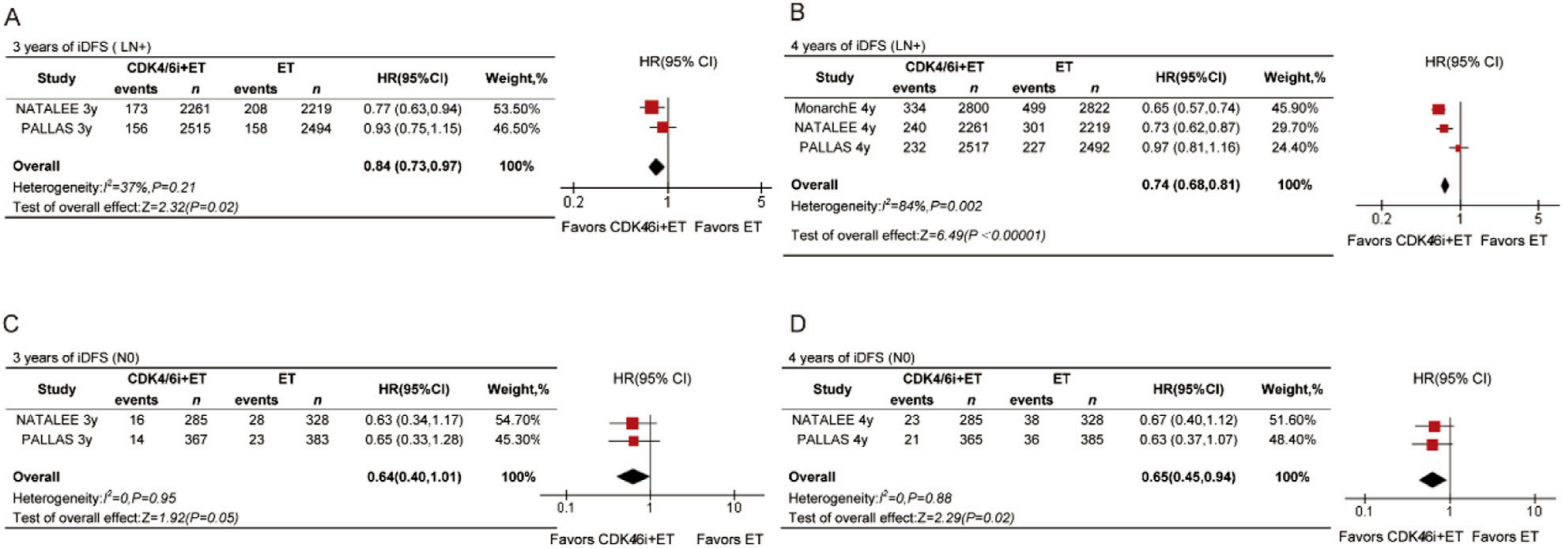
Forest plots of pooled hazard rations for 3-year iDFS and 4-year iDFS of CDK4/6 inhibitors plus ET versus ET in N0 **(A,B)** and N+ **(C,D)** patients.

For LN + patients, the data revealed statistically significant differences at both time points (Figures 4A,B). The 3-year HR for LN + patients receiving CDK4/6 inhibitors was 0.84 (95% CI 0.73–0.97, P = 0.02), and the 4-year HR was 0.74 (95% CI 0.68–0.81, P = 0.002). We also analyzed the 3-year and 4-year iDFS in N0 patients. There was no significant difference in 3-year iDFS between the treatment arms (HR = 0.64, 95% CI 0.40–1.01, P < 0.05, Figure 4C). However, a statistically significant improvement in 4-year iDFS was observed for N0 patients receiving CDK4/6 inhibitors compared to ET (HR = 0.65, 95% CI 0.45–0.94, P = 0.02, Figure 4D).

### Stage II and III

**FIGURE 5:**
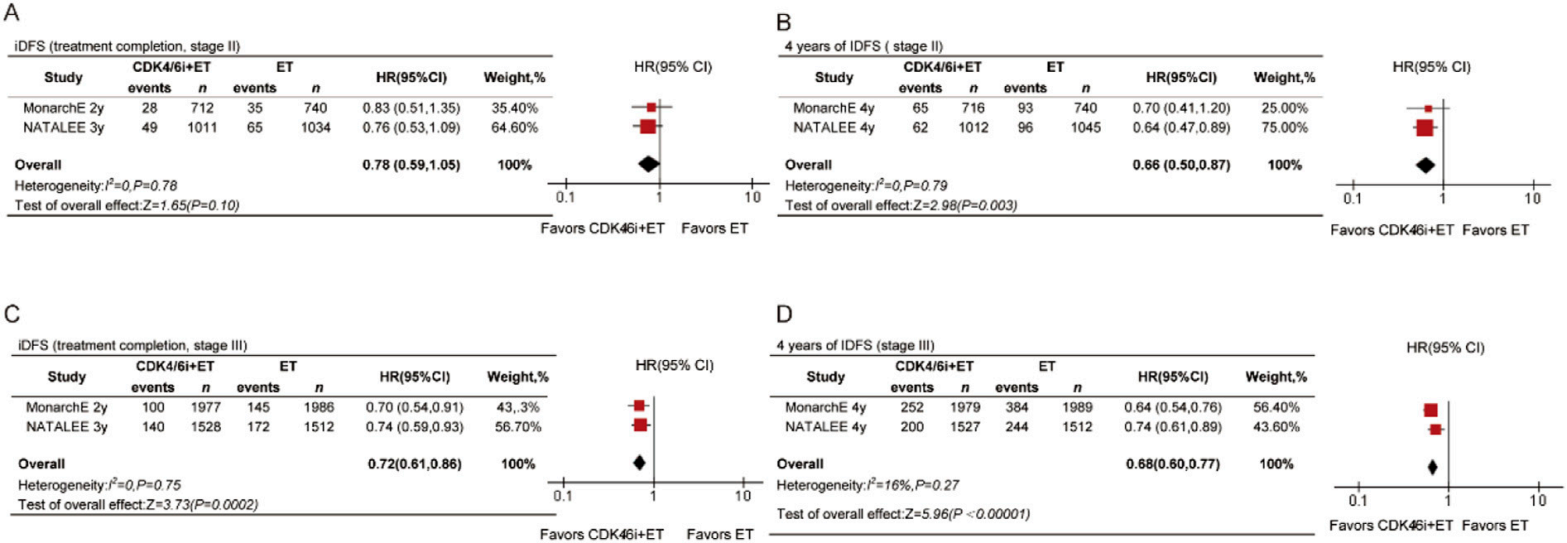
Forest plots of pooled hazard rations for iDFS at treatment completed and 4-year iDFS of CDK4/6 inhibitors plus ET versus ET in stage II **(A,B)** and stage III **(C,D)** patients.

To explore the impact of treatment duration, we included data on the time of treatment completion. iDFS showed statistically significant differences at both time points (Figures 5C,D) in stage III patients. At treatment completion, the HR was 0.72 (95% CI 0.61–0.86, P = 0.0002), and at 4 years, the HR was 0.68 (95% CI 0.60–0.77, P < 0.00001). The trend in benefits was not consistent for stage II patients. There was no significant difference in iDFS at treatment completion (HR = 0.78, 95% CI 0.59–1.05, P = 0.10, Figure 5A) in stage II patients. However, the results showed that the CDK4/6 inhibitors group achieved better iDFS compared with the ET group, with a statistically significant difference at 4 years for stage II patients (HR = 0.66, 95% CI 0.50–0.87, P = 0.003, Figure 5B).

## Discussion

Our data illustrate that the benefits of CDK4/6 inhibitors become more apparent with longer follow-up periods. In early-stage HR + patients, the incidence of survival events is typically low, necessitating extended time to adequately assess survival benefits. A study reported at ASCO evaluated the recurrence risk of HR+/HER2-early breast cancer treated with endocrine therapy (ET). The overall 3-year recurrence risk was 7%, and the 5-year recurrence risk was 12%. For N0 early breast cancer patients, the 3-year recurrence risk was 5%, while for N+ patients, it was 13% (Curigliano et al., 2024). Additionally, the sample sizes in each study are relatively small. The number of N0 patients in NATALEE and PALLAS is 285 and 365, respectively. Therefore, compared with N+ patients, sufficient time is required for the accumulation of invasive events in the N0 population. Extending the follow-up period allows for more events to occur, thereby providing a more accurate assessment of the therapy’s efficacy. These findings have important implications for future clinical trial designs, including patient selection and treatment duration, and highlight the need for long-term data from other RCTs.

For N0 and stage II patients, the benefit of CDK4/6i is partly attributed to inhibiting the long-term recurrence of HR + patients. The mechanism of CDK4/6 inhibitors can partly explain this phenomenon. ET is particularly important for HR + early breast cancer patients. However, ET resistance is associated with relapse in HR + patients, posing a significant challenge in treatment. Loss of function of NF1 is a mechanism of acquired resistance to endocrine therapy in breast cancer. Adding CDK4/6 inhibitors to ET was shown to overcome endocrine resistance driven by NF1 loss, synergistically enhancing ET, delaying and reversing endocrine resistance (Mendes-Pereira et al., 2012; Pearson et al., 2020). Previous studies have shown that early recurrence is more influenced by chemotherapy than by ET, which plays a crucial role in long-term survival (The Cancer Genome Atlas Network, 2012; Zardavas et al., 2013; Early Breast Cancer Trialists' Collaborative Group, 2005).

Our data supports the consideration of more intensive treatment, broader use of CDK4/6 inhibitors, for HR+, HER2- N0 patients and stage II patients. N+ status has traditionally been regarded as a significant risk factor for poor prognosis in early breast cancer, while N0 patients have received less consideration. This view is based on the previous prevailing model that distant metastases are seeded by lymph node metastases. In the SOFT-TEXT study, the 12-year overall survival for N0 patients reached 95.8%, while it was 89.7% for those with 1-3 positive lymph nodes, and 69.2% for patients with 4 positive lymph nodes after 5 years of exemestane + ovarian function suppression (Pagani et al., 2023). Nevertheless, recent studies investigating the evolutionary relationship between primary tumors, lymphatic and distant metastases revealed that lymphatic and distant metastases originated from independent subclones within the primary tumor, whereas, in some cases, they shared common subclonal origin (Naxerova et al., 2017). This study indicates that the primary tumor site can independently drive lymph node and distant metastasis. Consequently, the risk of metastasis does not depend solely on lymph node involvement, and high-risk patients should not be limited to those with positive lymph nodes.

Real-world data on breast cancer metastasis support the aforementioned findings. A study involving 1,520 patients examined metastatic risk in operable breast cancer and found that the metastatic proclivity of a tumor, in addition to the number of lymph nodes involved, increases with tumor size. (Heimann and Hellman, 2000). A recent real-world study explored the risk of recurrence and mortality in patients with HR+/HER2-early breast cancer, including N0 patients with high-risk features. The study included 7,564 HR+, HER2-early breast cancer patients with stage I to III disease, of which 5,557 were N0 patients, accounting for 73.4%. The results showed that the risk of recurrence and metastasis in high-risk N0 patients was almost similar to that in N1 patients, with 7-year follow-up rates of 16.9% and 17.1%, respectively. The 5-year recurrence rate was significantly higher in high-risk N0 patients (12.6%) compared to low-risk N0 patients (3.7%) (Jhaveri et al., 2024). These findings underscore the importance of monitoring both short- and long-term recurrence risks and providing intensive treatment for early-stage, high-risk breast cancer patients.

The NATALEE trial showed that invasive disease-free survival (iDFS) gradually increases with extended follow-up, rising from 2.7% at 3 years to 4.9% at 4 years, with the HR decreasing from 0.749 to 0.715. This indicates sustained benefits from ribociclib treatment beyond 3 years. Over time, the iDFS benefit of ribociclib combined with non-steroidal aromatase inhibitors continues to grow across different subgroups and secondary endpoints, particularly in patients with stage II/III and N0 or N+, showing a consistent trend of increasing efficacy. The MonarchE study’s 5-year results further expand iDFS from 6.4% at 4 years to 7.6% at 5 years. Our analysis also demonstrates a similar trend in overall populations, as well as in N+ and stage II/III populations, with the HR decreasing over time. As follow-up continues, we observe that HR+/HER2-patients continue to benefit from intensified treatment with CDK4/6 inhibitors. Even after completing the treatment, patients can still derive benefits from the drug. Ongoing follow-up is in progress, and we anticipate that CDK4/6 inhibitors will continue to reduce the risk of recurrence as follow-up time increases.

How to further reduce the risk of recurrence in patients with HR+/HER2-, N0 breast cancer is a hot topic in current clinical research. Our analysis shows that CDK4/6 inhibitors show promise in reducing recurrence risk for HR+/HER2-breast cancer patients, including those with node-negative (N0) disease. However, the standardized definition of high recurrence risk in N0 patients is still unclear, which makes it an urgent problem to screen who would benefit most from this treatment. The study of Luca Arecco (Arecco et al., 2025) provides some inspiration for this: compared with the MonarchE study, patients who only meet the high-risk definition of the NATALEE study show better prognosis. Is there a possibility that this group of people is overtreated? This finding suggests that for the N0 patient population, the true degree of clinical benefit and whether endocrine intensive treatment is needed still need to be further explored. In clinical practice, the safety issues brought about by long-term drug treatment cannot be ignored. For example, adverse reactions such as diarrhea symptoms associated with abemaciclib, hematological toxicity and cardiac toxicity caused by ribociclib, etc., will not only affect the quality of life of patients, but also may lead to decreased treatment compliance. This was confirmed in the NATALEE trial: about 29% of the patients in the experimental group discontinued ribociclib treatment for reasons other than disease progression. Although the overall toxicity of CDK4/6 inhibitors is controllable and well tolerated, it is still necessary to optimize treatment compliance through adequate patient education and standardized follow-up management. In addition, cost is also an important consideration in clinical decision-making. A comment pointed out that the treatment plan may cost about $15 million to save the life of one patient (Slamon et al., 2024), which obviously exceeds the conventional cost-effectiveness threshold and may impose a heavy burden on the medical system. Therefore, while expanding the population that can benefit from CDK4/6 inhibitors, it is crucial to accurately identify high-risk N0 patients who may really benefit, avoiding blind expansion of the treatment population. Of course, optimizing drug pricing through negotiations between the government and pharmaceutical companies may be a potential way to solve this problem.

This study has several limitations. As there is no available detailed data, when extracting data, such as the MonarchE 3-year and the Penelope-B 4-year, the Engauge Digitizer tool was used to extract information from survival curves extract. Due to image resolution, manual point selection, curve overlap, etc., there will be systematic differences between the extracted survival rate values and the true values, which may lead to measurement bias in the analysis results. Some conclusions drawn from the data exhibit high heterogeneity, (e.g., 3-year and 4-year IDFS), prompting the use of a random-effects model. The observed heterogeneity likely stems from variations in study populations, treatment regimens, and study designs across the included trials. Population included differed substantially among trials. The MonarchE study included more than 4 positive axillary lymph nodes, or 1-3 positive nodes plus additional high-risk features. PALLAS included Stage II-III breast cancer. NATALEE Included both N0 patients (stage IIA with specific features) and N1+, IIB-III stage patients. Penelope-B included HR + patients with residual disease after neoadjuvant chemotherapy. The definition of “high-risk” patients in various clinical trials is significantly different. In terms of treatment regimen, the studies employed different CDK4/6 inhibitors: Abemaciclib (MonarchE), Palbociclib (PALLAS/Penelope-B), Ribociclib (NATALEE). Notably, NATALEE used a 3-year treatment duration versus 2 years in other trials. In terms of study design, except for Penelope-B, which used a double-blind design, the other studies were open-label trials. Due to the limited number of eligible studies, we could not perform comprehensive subgroup analyses for all potential heterogeneity sources. However, our analysis revealed consistently low heterogeneity in lymph node-negative and stage II-III subgroups, suggesting that the primary source of variation may come from differences in the definition of “high risk” for lymph node-positive patients. This finding emphasizes the importance of standardized definition of high-risk populations and provides an important reference for future study design. More extensive and higher-quality data in the future is required to validate our findings.

## Conclusion

In conclusion, adjuvant CDK4/6 inhibitors plus ET were associated with a reduced risk of invasive disease recurrence, as shown in the updated analysis. The benefit appeared to strengthen with longer follow-up times. These results suggest that CDK4/6 inhibitors may benefit a broader population and enhance the long-term efficacy for HR+, HER2-breast cancer patients with N0 or Stage II. Further research is needed to confirm this long-term efficacy. The survival benefit of CDK4/6 inhibitors may be partially mediated by reduced late recurrence, but additional studies are required to elucidate the underlying mechanisms.

## Data Availability

The original contributions presented in the study are included in the article/supplementary material, further inquiries can be directed to the corresponding author.
